# Supplementary material for: C-C Motif Chemokine Ligand 2 Enhances Macrophage Chemotaxis, Osteogenesis, and Angiogenesis during the Inflammatory Phase of Bone Regeneration
Source: Biomolecules. 2023 Nov 18;13(11):1665. doi: 10.3390/biom13111665 (PMC10669364; doi:10.3390/biom13111665)
Supplement: Supplementary file 1 [file biomolecules-13-01665-s001.zip › biomolecules-2673675-supplementary.pdf]

Supplementary information

Title: C-C Motif Chemokine Ligand 2 Enhances Macrophage Chemotaxis, Osteogenesis, and Angiogenesis during the Inflammatory Phase of Bone Regeneration

Supplemental figure S1. Indirect co-cultures with MSCs using transwell.

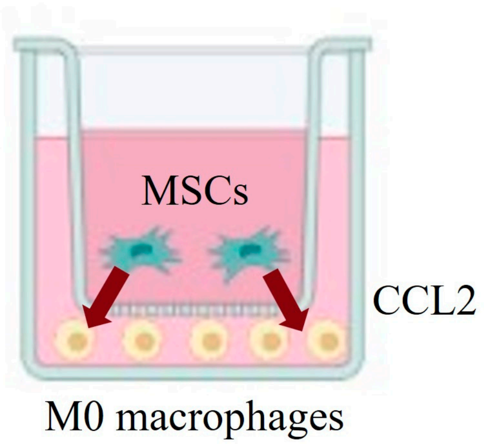

To evaluate the function of CCL2 secreted by MSCs, MSCs of each group were seeded on top of the transwells after seeding the macrophages.
